# Study on recognition of coal and gangue based on multimode feature and image fusion

**DOI:** 10.1371/journal.pone.0281397

**Published:** 2023-02-09

**Authors:** Lijuan Zhao, Liguo Han, Haining Zhang, Zifeng Liu, Feng Gao, Shijie Yang, Yadong Wang

**Affiliations:** 1 School of Mechanical Engineering, Liaoning Technical University, Fuxin, China; 2 Liaoning Provincial Key Laboratory of Large-Scale Mining Equipment, Fuxin, China; 3 Shandong Yankuang Group Changlong Cable Manufacturing Co., Ltd, Jining, China; PDPM IIITDM: PDPM Indian Institute of Information Technology Design and Manufacturing Jabalpur, INDIA

## Abstract

Aiming at the problems of low accuracy of coal gangue recognition and difficult recognition of mixed gangue rate, a coal rock recognition method based on modal fusion of RGB and infrared is proposed. A fully mechanized coal gangue transportation test bed is built, RGB images are obtained by camera, and infrared images are obtained by industrial microwave heating system and infrared thermal imager. the image data of the whole coal, whole gangue, and coal gangue with different gangue mixing as training and test samples, identify the released coal gangue and its mixing rate. The AlexNet, VGG-16, ResNet-18 classification networks and their convolutional neural networks with modal feature fusion are constructed. results: The classification accuracy of ResNet networks on RGB and infrared image data is higher than AlexNet and VGG-16 networks. The early convergence network performance of ResNet is verified through the convergence of different models. The recognition rate of the network is 97.92 the confusion matrix statistics, which verifies the feasibility of the application of modal fusion method in the field of coal gangue recognition. The fusion of modal features and early models of ResNet coal gangue, which is the basic premise for realizing intelligent coal caving.

## 1. Introduction

Fully mechanized mining and top coal mining have become two of the main methods of thick coal seam mining in China, and intelligent coal mining is the only way to the high-quality development of the coal industry [1, 2]. The basic premise of intelligent coal mining is to achieve intelligent coal caving, which must have the capability of automatic recognition of gangue. Coal gangue recognition is the key technology of fully mechanized mining and top coal mining and has a significant impact on the coal extraction rate and coal recovery quality. In recent years, many experts and scholars have conducted a lot of in-depth research on the coal gangue recognition problem. Liu Wei et al. [3] proposed a method for detecting the interface of coal gangue to identify the Hilbert spectrum information entropy feature of the vibration of coal gangue. Zhang Ningbo et al. [4] proposed a method for measuring and identifying the mixed coal gangue of coal gangue in the process of natural coal discharge, providing a basis for judging the appearance of coal gangue using natural coal radiation technology. Liu Chuang [5] [Unpublished] proposed an active gangue recognition method for microwave heating-infrared (IR) detection, studied the recognition mechanism, and analyzed its feasibility. Yuan Yuan et al. [6] designed a feature extraction and classification method for coal acoustic signals based on the wavelet packet decomposition and random forest (RF) algorithm. Dou Xijie et al. [7] proposed a gangue recognition method based on an intrinsic mode function energy moment and support vector machine, and this method effectively recognized the data of multiple gangue vibration samples. Xue Guanhui et al. [8] proposed a coal gangue image recognition method based on the RF algorithm. The RF model’s performance improved after dimension reduction. Jiang Lei et al. [9] based on convolutional neural networks (CNNs) and lightweight dilated CNNs, established a fully dilated CNN-based coal and coal gangue intelligent recognition model, using the hydraulic support tail beam vibration signal Mel frequency cepstral coefficient feature matrix as the CNN input. They realized the structure optimization of the recognition model, significantly improved the operation speed, reduced the use of resources, and revealed the recognition mechanism and classification basis of the model. Shan Pengfei et al. [10] investigated the optimization method of the attention mechanism fusion in the ResNet50 backbone feature extraction network, which determined the best fusion position with the coal-gangue falling state detection as the target, increasing the ability to extract the weight information of coal-gangue. Zhang Jinwang et al. [11] proposed a new concept of coal gangue recognition of "liquid intervention + infrared detection," and a recognition test of liquid intervention under different mixing degrees was conducted. By selecting a reasonable liquid type, temperature, and intervention amount, the coal rock recognition accuracy can be effectively improved. Combined with the virtual prototype technology, a project team proposed a coal rock cut state recognition scheme based on the cyber-physical system concept and integrated heterogeneous data such as acquisition, processing, and recognition data from multiple fields, realizing the adaptive height adjustment of the shearer [12, 13]. Due to the poor working conditions and complex environment, the occurrence conditions of the top coal, the coal discharge mode of the tail beam, the kinematic parameters of the scraper conveyor, the gradient characteristics of the hydraulic system, and the interaction between the roof beam and roof will directly or indirectly affect the top coal caving process of the top coal caving support under the conditions of existing coal gangue. Although the recognition technology of integrated gangue based on vibration and sound characteristics can realize the recognition of gangue, available signals can be easily polluted by environmentally stimulated signals during the underground production process; thus, the ability to effectively extract gangue signals from the mixed signals is critical [14] [Unpublished]; Although research on natural radiation methods and other technologies is not limited by the underground coal discharge environment, it is difficult to apply them to a working face that does not contain radioactive elements or has a low content of radioactive elements and contains too much gangue in coal gangue [15] [Unpublished]. Therefore, how to quickly and accurately identify the rapidly moving coal gangue and its gangue content on a rear scraper conveyor remains a technical bottleneck to realizing intelligent coal discharge. The image-based recognition method of coal gangue primarily obtains information about the coal caving status using cameras based on the coal caving mechanism and employs machine learning algorithms to complete the recognition and classification task, which have the advantages of high reliability and fast response. However, the fast movement of back scraper conveyors carrying coal gangue, the low brightness of the underground environment, and the high dust level remain some of the technical bottlenecks of using images to identify coal gangue and realize intelligent coal caving.

In this study, a multimodal information acquisition and test platform of gangue microwave excitation is constructed. By simulating the motion state of a scraper conveyor at the back of the working face, the RGB image data of gangue are obtained by cameras, and the IR image data of gangue are collected by an IR thermal imager. A two-input CNN based on ResNet is constructed, and the modal fusion of RGB and IR images by feature fusion is adopted to realize the accurate recognition of gangue and gangue content, verify the feasibility of the modal fusion method in the field of gangue recognition, and provide a new method for intelligent coal release. Accurate recognition of coal gangue is achieved by combining multimodal features and ResNet model technology, which replaces the eye recognition of coal miners, improves mining safety, increases the top coal extraction rate, and provides a new method for intelligent coal caving and coal mine intellectualization.

## 2. Research methods

In recent years, modal fusion techniques have been extensively used in medical imaging omics [16–18], self-driving [19], mass detection [20], and other fields. Combined with the characteristics of coal gangue recognition in a comprehensive discharge working face, mode fusion technology is applied to research on intelligent top discharge coal discharge support with low illumination, high dust, and small space. Through torch.cat channel splicing, the features of gangue RGB and IR images are integrated, a multimodal fusion network based on ResNet-18 is constructed, and the CrossEntropyLoss loss function is used for gangue classification while improving the recognition accuracy and ensuring system stability.

### 2.1 Convolutional neural network

A CNN is one of the representative algorithms of deep learning, which introduces the local connection and weight sharing between the network layers, reducing the model parameters and solving the problems of difficult convergence and overfitting. A CNN is very suitable for image processing. During each convolution operation, an image with an input size of *H*_*i*_ * W_*i*_ is subjected to a convolution kernel of *F*_*h*_ * F_*h*_. The dilated convolution value is *D*, the filling is *P*_*h*_ * P_*h*_, and the step size is *S*_*h*_ * S_*w*_. The output image size *H*_*o*_ * W_*o*_ is as follows:

$$H_{o}=[\frac{H_{i}+2\times P_{h}-D\times (F_{h}-1)-1}{S_{h}}+1],$$ (1)

$$W_{o}=[\frac{W_{i}+2\times P_{w}-D\times (F_{w}-1)-1}{S_{w}}+1].$$ (2)

**Fig 1:**
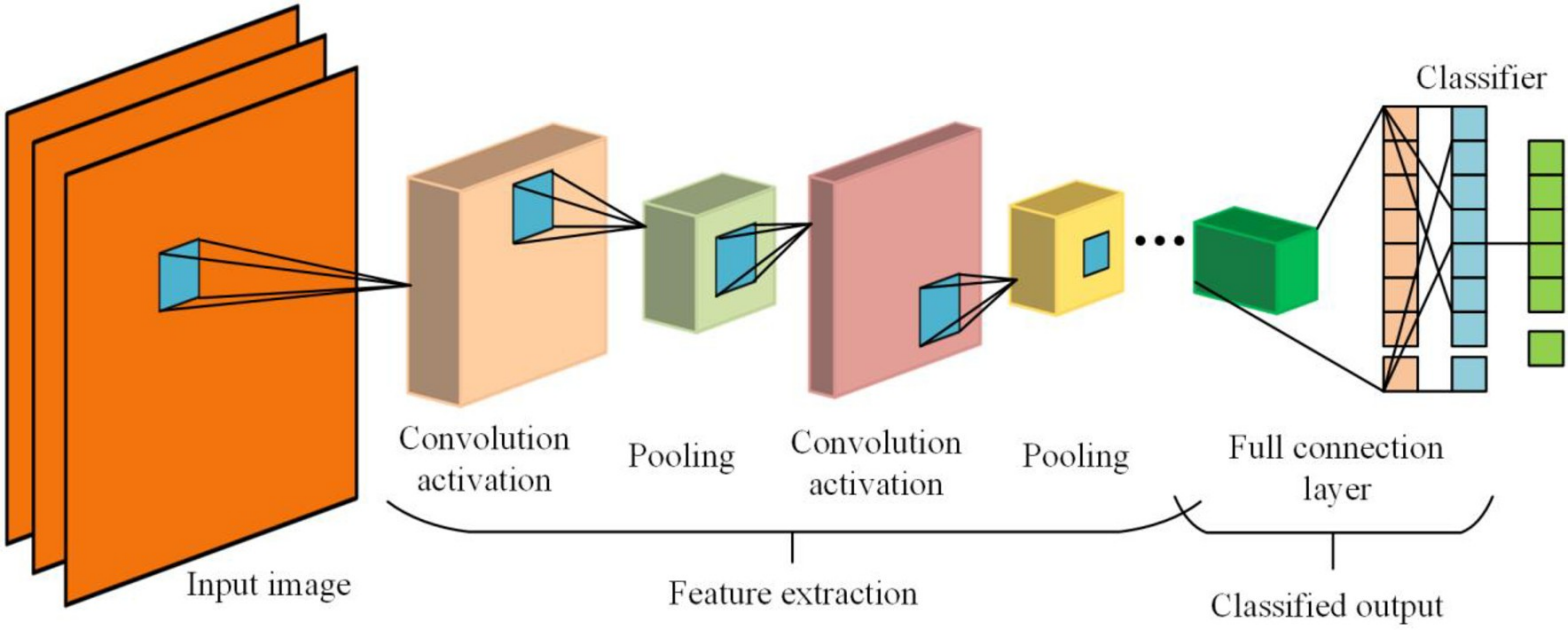
CNN structure.

The main structure of the CNN includes the convolutional, pooling, and fully connected layers, and a typical CNN classification network structure is depicted in Fig 1.

### 2.2 Modal fusion method based on ResNet

**Fig 2:**
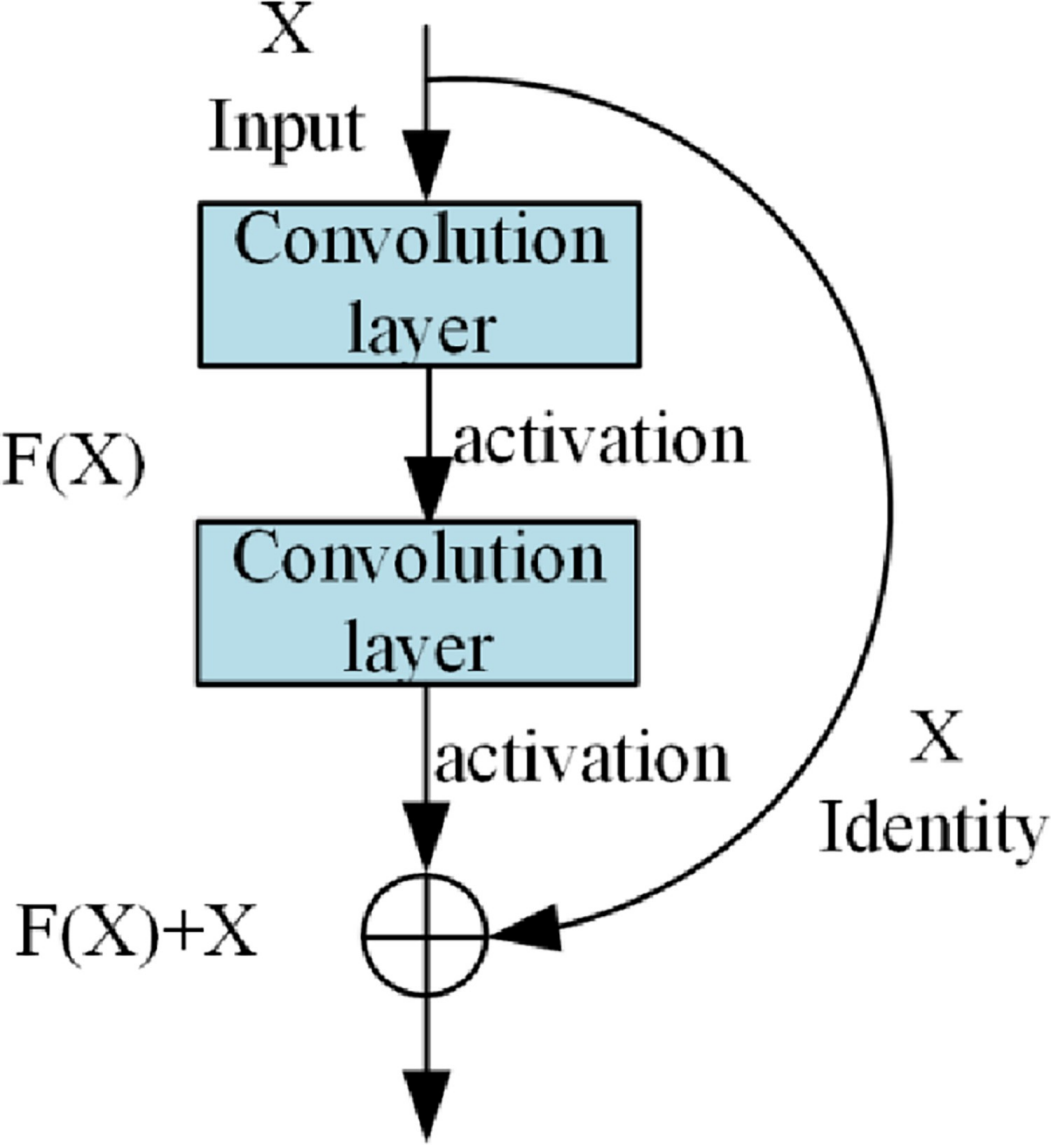
The ResNet residual module.

**Fig 3:**
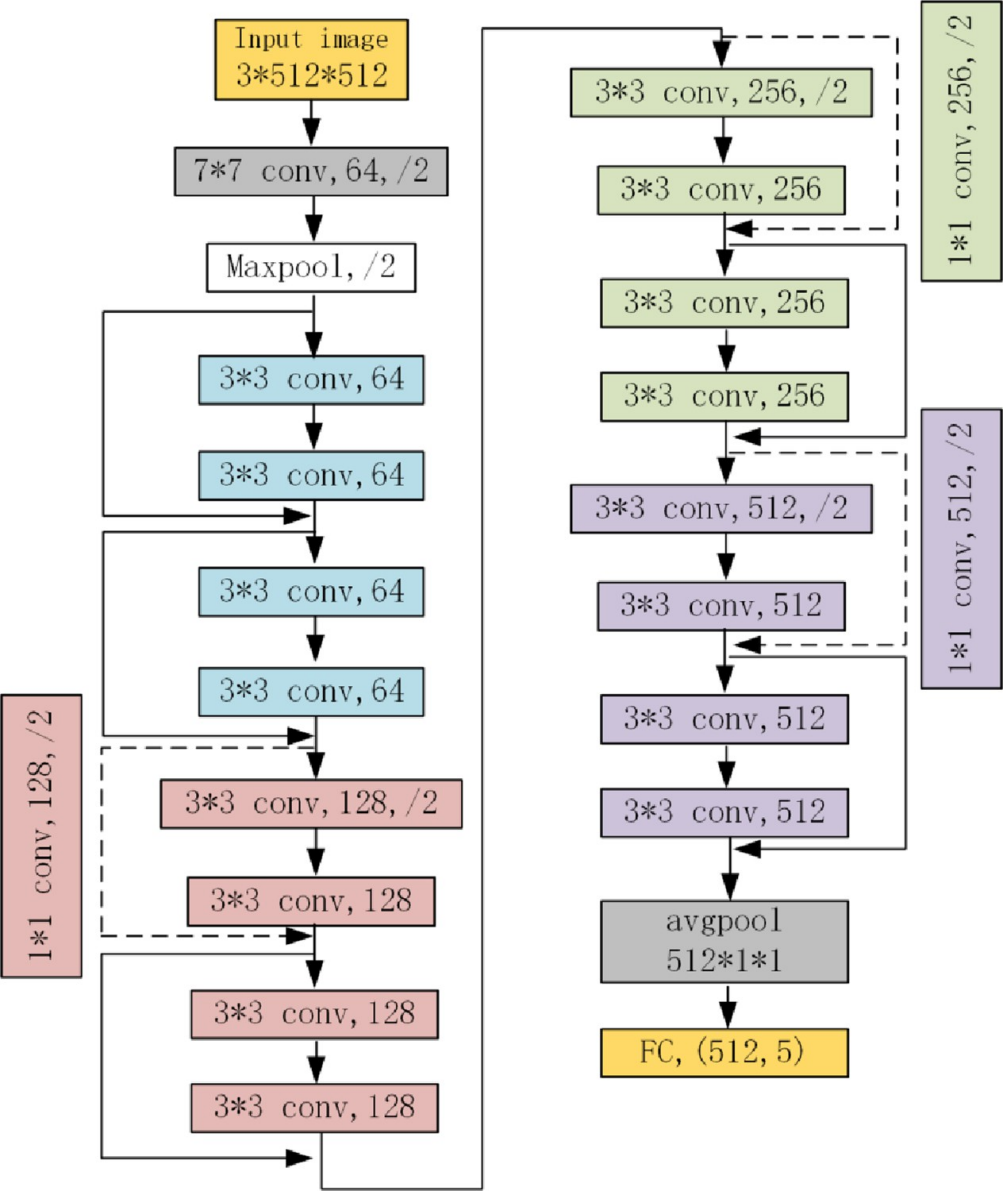
ResNet-18 network structure diagram.

The deep residual network (DRN) was launched in 2015 [21, 22]. Compared with traditional deep neural networks, a jump connection mode is used in DRN, which eliminates harmful data features and solves the problem of error gradient disappearance with an increase in network depth [23]. Taking the classical ResNet-18 network, as an example, it is composed of multiple residue blocks, one of which is shown in Fig 2, and the network structure is depicted in Fig 3.

**Fig 4:**
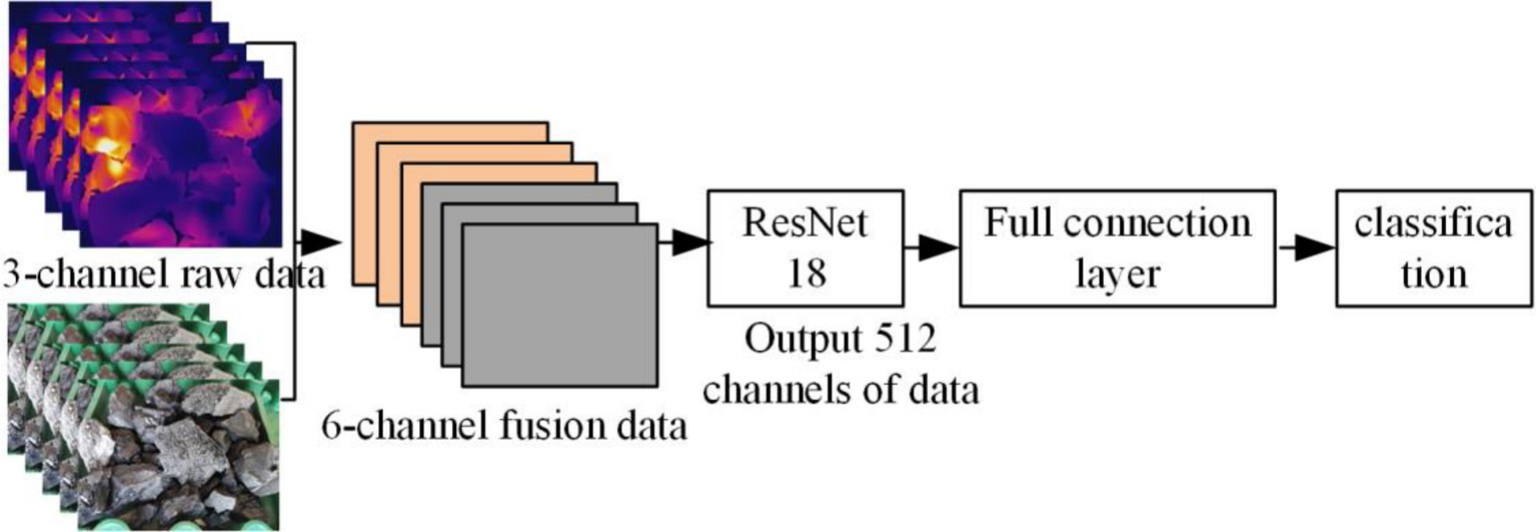
The early fusion network.

**Fig 5:**
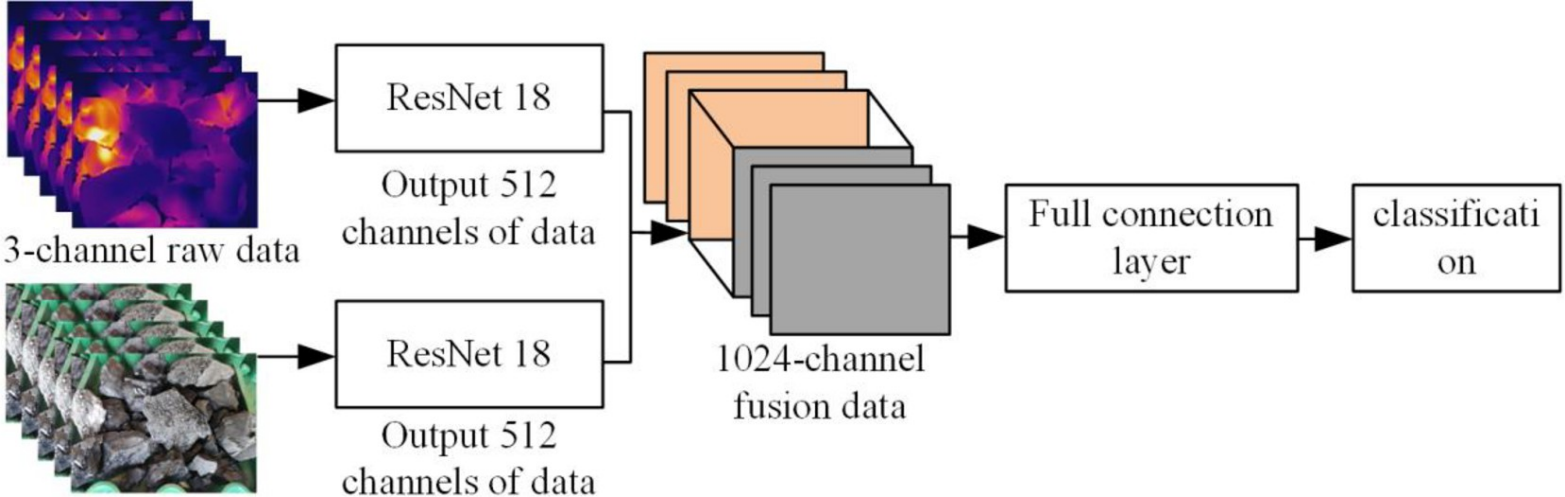
Late fusion network.

Modal data include visible light, IR, and depth images [24, 25]. According to the data level, the modal fusion mode can be divided into pixel-level fusion, feature-level fusion, and decision-level fusion. In this study, a feature-based mode fusion scheme is proposed, and an early mode fusion method is constructed. The model structure is depicted in Fig 4. The pseudocode of the ResNet early fusion algorithm is shown in Table 1. The two types of original mode data with three channels are fused into six-channel data that are input into the ResNet network for training to accurately obtain the corresponding category by torch.cat channel splicing. The model structure of late mode fusion is shown in Fig 5. The pseudocode of the ResNet late fusion algorithm is shown in Table 2. The original modal data of two types of three channels are passed through the ResNet network to form 512 channels of data, which are then spliced into 1024 channels by torch.cat. Finally the data is trained through a fully connected layer to obtain the accuracy of the corresponding category. In addition, the RGB and IR image data of coal gangue are input to the network in one-to-one correspondence. The RGB and IR image data names of coal gangue at the same time have the same part. By retrieving the name of the same part, the data at the same time will be input into the network one by one. The feature-based mode fusion method can reduce the heterogeneity difference between modes while maintaining the integrity of specific features of each mode, effectively overcoming the problem of a low single-mode recognition rate and significantly improving the generalization ability of the model.

**Table 1:** ResNet early fusion pseudocode.

**Algorithm 1:** ResNet early modal feature fusion algorithm
**Input:** Infrared dataset **D**_**h**_, RGB dataset **D**_**z**_, Dataset label **L**, ResNet model *M*, Iteration number *N*;
**Output:** Accuracy of image classification of test set;
1 Divide the data set {**D**_**h**_**, D**_**z**_**, L**} into training set {**D**_**h1**_**, D**_**z1**_**, L**_**1**_} and test set {**D**_**h2**_**, D**_**z2**_**, L**_**2**_}; The corresponding batch is divided into **B**_**1**_ and **B**_**2**_;
2 **for** *j* = 0:*N*
3 **foreach** *b*_*1*_∈**B**_**1**_
4 Fusion of training data on channel dimension, **D**_**1**_**_b**_**1**_ = concat (**D**_**h1**_**_b**_**1**_**, D**_**z1**_**_b**_**1**_);
5 Send **D1_b1** to model *M* for training;
6 **foreach** *b*_*2*_∈**B**_**2**_
7 Fusing the test data on the channel dimension, **D**_**2**_**_b**_**2**_ = concat (**D**_**h2**_**_b**_**2**_**, D**_**z2**_**_b**_**2**_);
8 Test model *M;*
9 Get Accuracy

**Table 2:** ResNet late fusion pseudocode.

**Algorithm 2:** ResNet late modal feature fusion algorithm
**Input:** Infrared dataset **D**_**h**_, Natural light data set **D**_**z**_, Dataset label **L**, ResNet model about *M*_*h*_ and *M*_*z*_ without full connection layer FC1, Iteration number *N*;
**Output:** Accuracy of image classification of test set;
1 Divide the data set {**D**_**h**_**, D**_**z**_**, L**} into training set {**D**_**h1**_**, D**_**z1**_**, L**_**1**_} and test set {**D**_**h2**_**, D**_**z2**_**, L**_**2**_}; The corresponding batch is divided into **B**_**1**_ and **B**_**2**_;
2 **for** *j* = 0:*N*
3 **foreach** *b*_*1*_∈**B**_**1**_
4 Training model *M*_*h*_ **D**_**h1**_**_b**_**1**_**_f** = *M*_*h*_(**D**_**h1**_**_b**_**1**_);
5 Training model *M*_*z*_ **D**_**z1**_**_b**_**1**_**_f** = *M*_*z*_(**D**_**z1**_**_b**_**1**_);
6 Fusion of two modal features in channel dimension **D**_**1**_**_b**_**1**_**_f** = concat(**D**_**h1**_**_b**_**1**_**_f**, **D**_**z1**_**_b**_**1**_**_f**);
7 Send **D**_**1**_**_b**_**1**_**_f** to the full connection layer FC2 for testing;
8 **foreach** *b*_*2*_∈**B**_**2**_
9 Dh2_b2_f = Mh(Dh2_b2);
10 Dz2_b2_f = Mz(Dz2_b2);
11 Fusion of two modal features in channel dimension **D**_**2**_**_b**_**2**_**_f** = concat(**D**_**h2**_**_b**_**2**_**_f**, **D**_**z2**_**_b**_**2**_**_f**);
12 Send **D**_**2**_**_b**_**2**_**_f** to the full connection layer FC2 for testing;
13 Get Accuracy

### 2.3 Loss function and evaluation index

In PyTorch, for multiclassification problems, nn.Crossentropyloss is used as the loss function, which calculates the cross entropy loss between the predicted value and the target. Here, the CrossEntropyLoss loss function is applied, and it is no longer necessary to use the Softmax classifier for the probability mapping of input features. The memory network input is (*N*, *C*), where N represents the minibatch number, and C represents the total number of categories. Its loss function is defined by the following Eq (3):

$$l_{n}=-\log\frac{\exp(x_{n,y_{n}})}{\sum_{c=1}^{C}\exp(x_{nc})},$$ (3)

where *x*_*nc*_ denotes the output value of the network, *n* = 1, 2,…, *N*, *c* = 1, 2, …, *C*, and the true category of the nth sample is *y*_*n*_.

In the actual sample acquisition process, there may be data imbalance between different categories. Therefore, it is necessary to introduce a weight *K* = (*k*_*1*_, *k*_*2*_, *…*, *k*_*C*_) to ensure the data balance; the loss function after adding the weight is defined by Eq (4):

$$l_{n}=k_{y_{n}}(-x_{n,y_{n}}+\log(\sum_{c=1}^{C}\exp(x_{nc}))).$$ (4)

The actual network training is conducted in minibatch units, and the loss after an iterative training session can be returned in three ways, as shown in Eq (5):

$$l=\{\begin{array}{l} (l_{1},l_{2},\cdots ,l_{N})\;\mathrm{if}\;\mathrm{reduction}="\mathrm{none}"\\ \frac{\sum_{n=1}^{N}l_{n}}{\sum_{n=1}^{N}k_{yn}}\;\mathrm{if}\;\mathrm{reduction}="\mathrm{mean}"\\ \sum_{n=1}^{N}l_{n}\;\mathrm{if}\;\mathrm{reduction}="\mathrm{sum}" \end{array}.$$ (5)

In the accuracy evaluation of network models, accuracy (ACC), precision (PPV), recall rate (TPR), and F_1_ score are typically used to measure the performance of recognition networks [26]. ACC indicates the proportion of the number of samples in the total samples correctly classified as coal gangue, which can be determined by Eq (6).


$$ACC=\frac{TP+TN}{TP+TN+FP+FN}.$$ (6)

PPV represents the weighted average value of the precision (PPV_i_) of different coal gangue categories, revealing the discrimination ability of the recognition network to negative samples, which can be determined by Eqs (7) and (8). PPV_i_ is proportion of correct numbers predicted to be in that category versus all predicted to be in that category among the gangue prediction category of the classification network model, where *i* indicates a category, *N* represents the total number of categories, and *K* indicates the weight of a gangue sample in the total sample.


$$PPV_{i}=\frac{TP_{i}}{TP_{i}+FP_{i}},$$ (7)

$$PPV=\frac{\sum_{i}^{N}PPV_{i}\times K_{i}}{N}.$$ (8)

TPR represents the weighted average value of different coal gangue categories (TPR_i_), revealing the discrimination ability of the recognition network to positive samples, which can be determined by Eqs (9) and (10). Here, *i* indicates a category in the classification network model and TPR_i_ denotes the ratio of the number of samples correctly predicted to the total number of samples in that category.


$$TPR_{i}=\frac{TP_{i}}{TP_{i}+FN_{i}},$$ (9)

$$TPR=\frac{\sum_{i}^{N}TPR_{i}\times K_{i}}{N}.$$ (10)

F_1_ score, as the weighted average of precision (PPV) and recall (TPR), is used to evaluate the classification network performance and can be determined by Eq (11).


$$F_{1}=\frac{2PPV\times TPR}{PPV+TPR}.$$ (11)

## 3. Simulation test of coal gangue movement state

In the process of gangue identification, the accurate acquisition of multimodal features is a prerequisite for improving the speed and accuracy of gangue identification. Because gangue infrared features were more obvious gangue RGB features after excitation by a heat source, a microwave-heated gangue transport test bench was constructed in this section. Both RGB and infrared images of gangue were acquired simultaneously. Multimodal features of gangue were obtained and preprocessed by scientifically setting the mixing rate and designing multiple groups of test categories.

### 3.1 Construction of test bench

**Fig 6:**
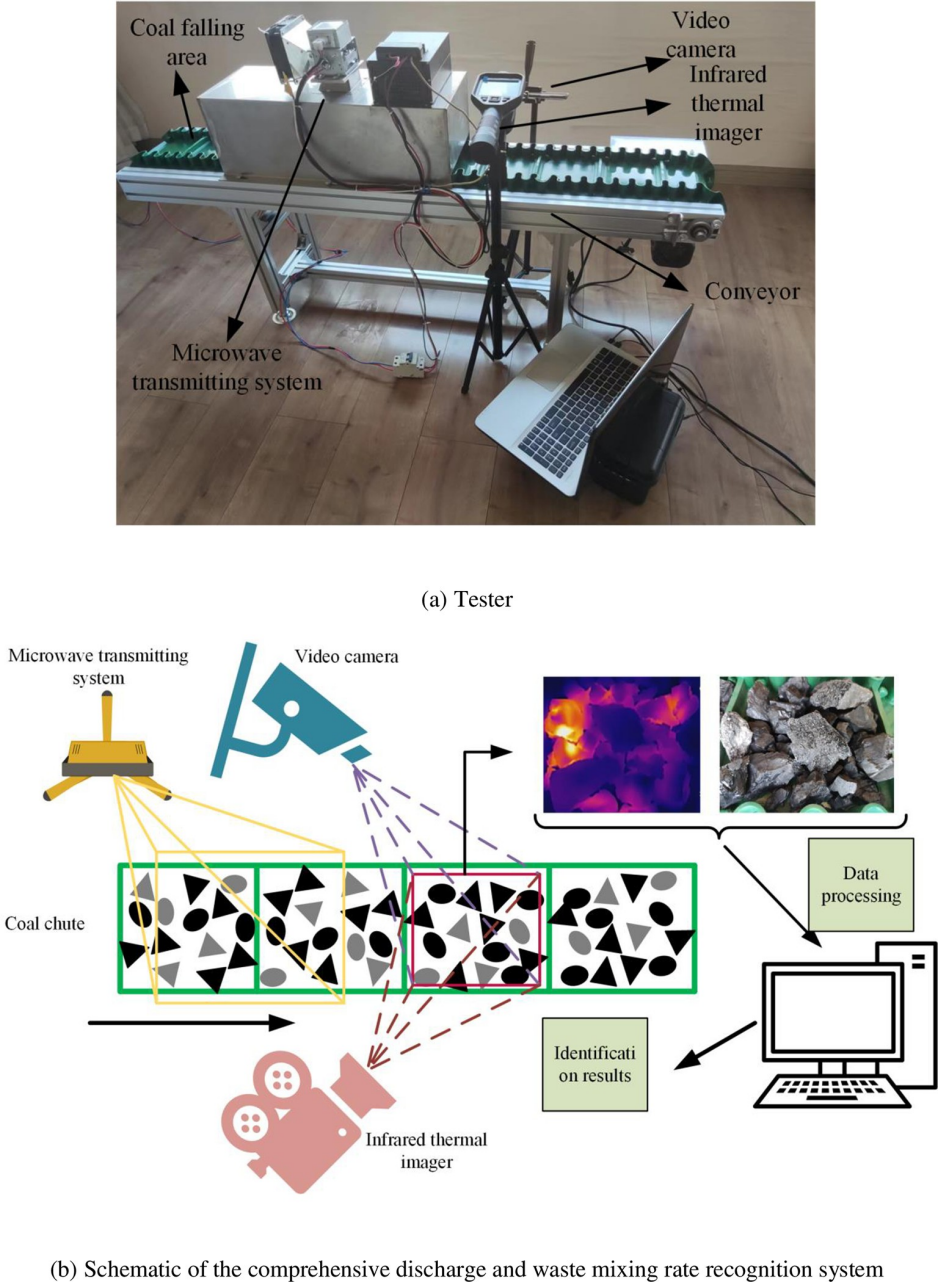
Multimodal image acquisition test platform. (a)Tester; (b) Schematic of the comprehensive discharge and waste mixing rate recognition system.

A multimodal information acquisition test platform of coal gangue microwave excitation is constructed in this study. By simulating the movement state of the scraper conveyor at the back of the fully mechanized top coal caving face, RGB and infrared images excited by microwave are collected. The experimental device is primarily composed of the main conveyor, industrial-grade microwave emission system, RGB image collector, and infrared thermal imager. A schematic of the acquisition equipment and recognition system is depicted in Fig 6. The microwave emission system is primarily composed of a magnetron, waveguide, thermostat, fan, air guide, power supply, and shielding shell. The RGB image collector consists of a triangle holder and a video camera. The resolution of the image taken by the camera on the computer is 720*1280, and the number of frames taken per second is 30 fps. The Haikang Micro Shadow Infrared Thermal Imager under Haikang Video H16 was used in this experiment. Its output image resolution on a computer is 640*480 pixels, and the number of frames taken per second is 25 fps.

### 3.2 Test scheme

Gangue content in the coal release process [27] can be determined using Eq (12):

$$R=\frac{M_{r}}{M_{c}+M_{r}}\times 100\%.$$ (12)

Here, *M*_*c*_ denotes the volume of the top coal, and *M*_*r*_ denotes the volume of the gangue.

**Fig 7:**
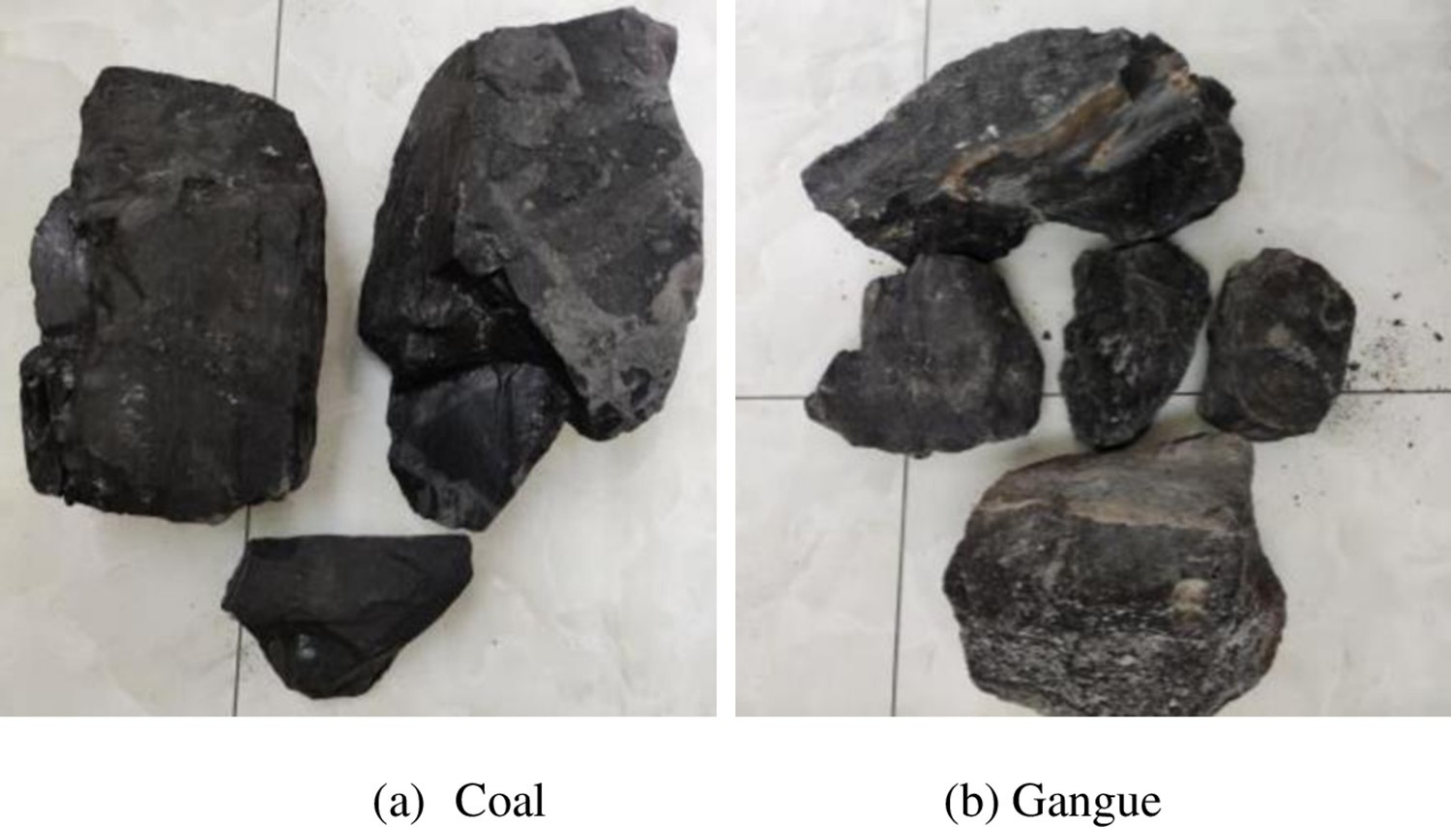
Coal gangue test sample. (a) Coal; (b) Gangue.

**Fig 8:**
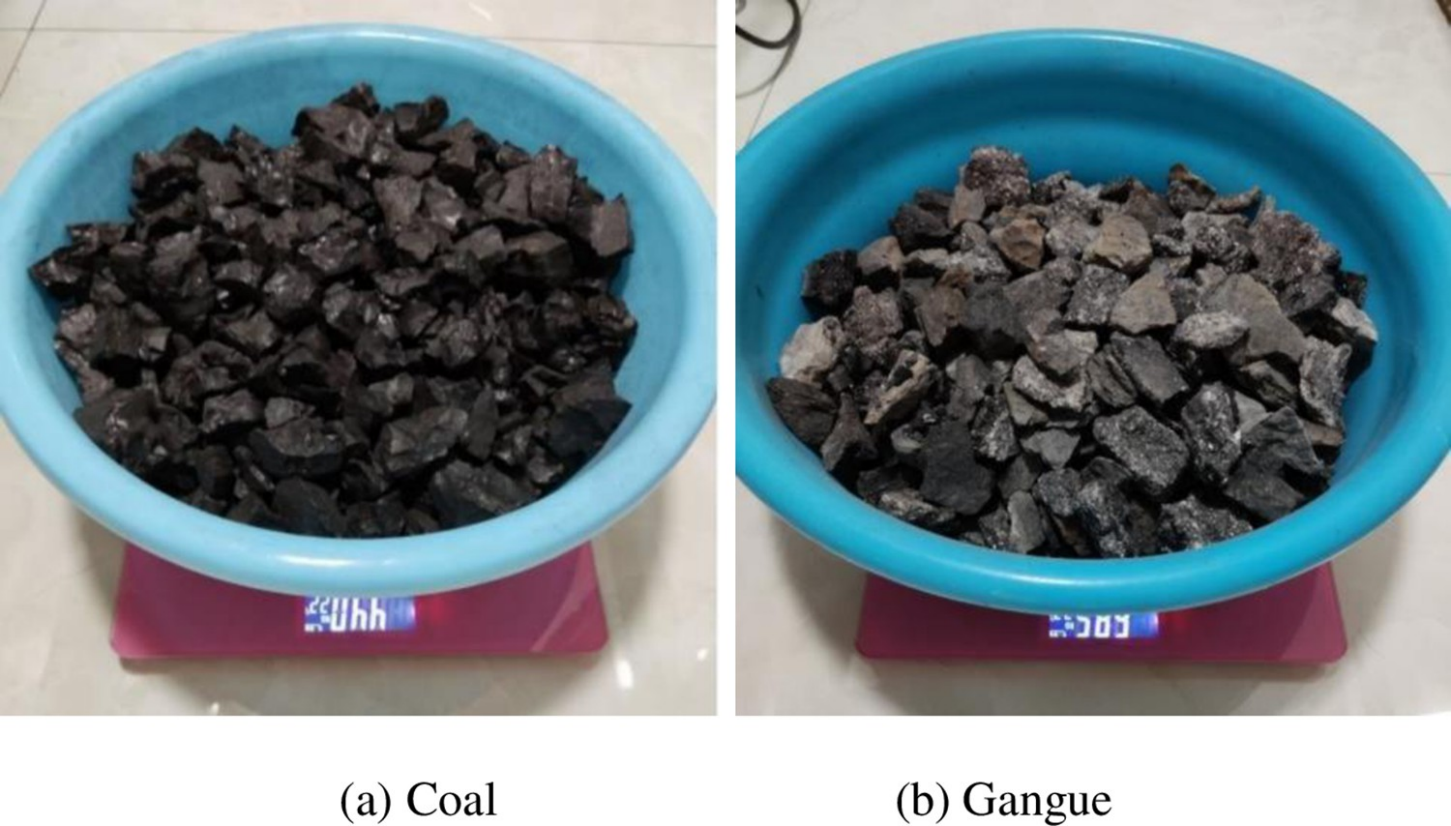
Mass measurement of coal gangue. (a) Coal; (b) Gangue.

**Fig 9:**
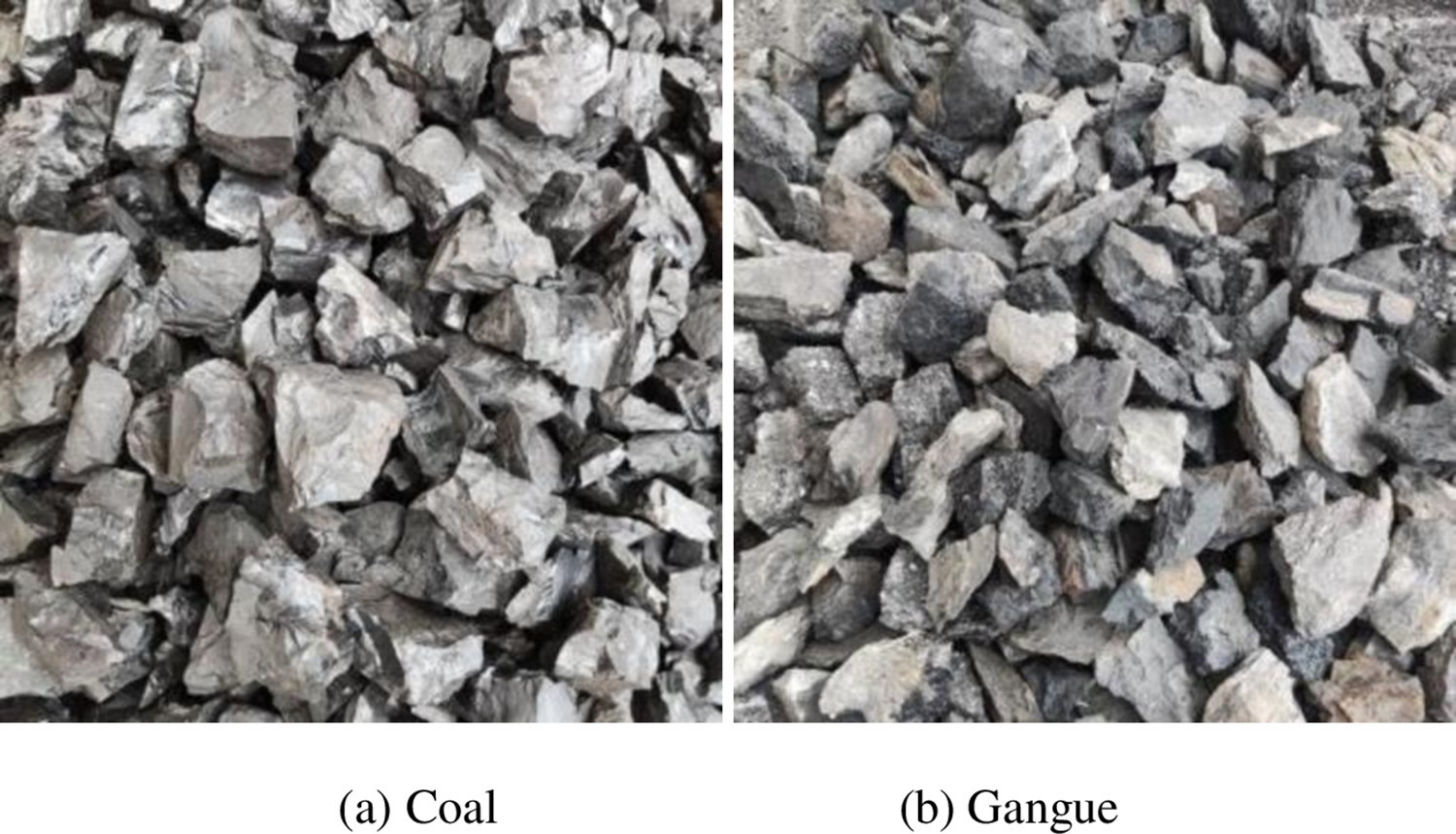
Broken coal gangue sample. (a) Coal; (b) Gangue.

The original coal gangue sample is shown in Fig 7. The mass of the coal gangue sample is measured by electronic scale, as depicted in Fig 8. The samples are divided into whole coal, whole gangue, 10% gangue content [28, 29], 25% gangue content [30], and 50% gangue content [30]. The five groups were tested, as shown in Fig 9.

### 3.3 Data acquisition and production

**Fig 10:**
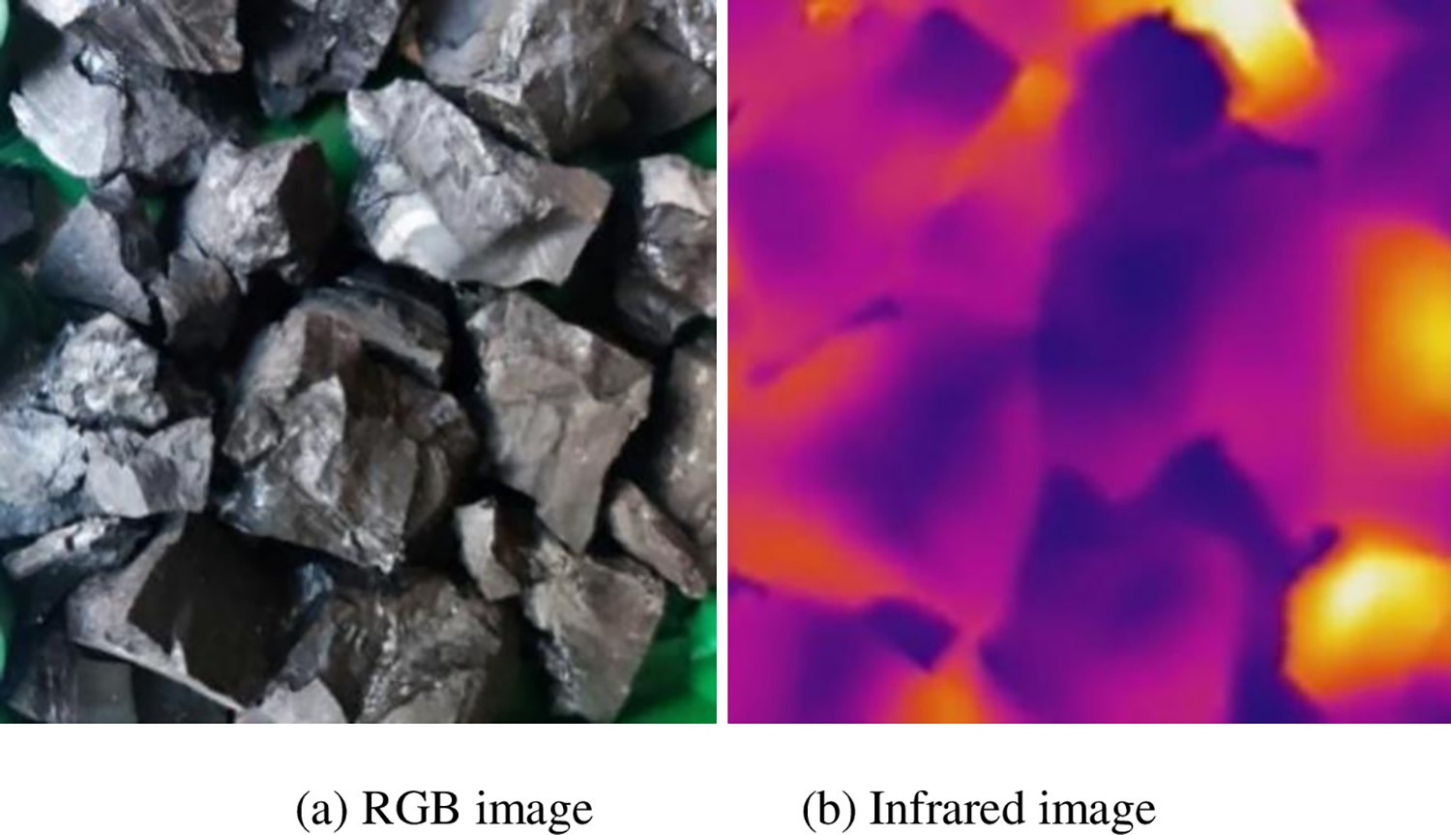
Multimodal image of whole coal. (a) RGB image; (b) Infrared image.

**Fig 11:**
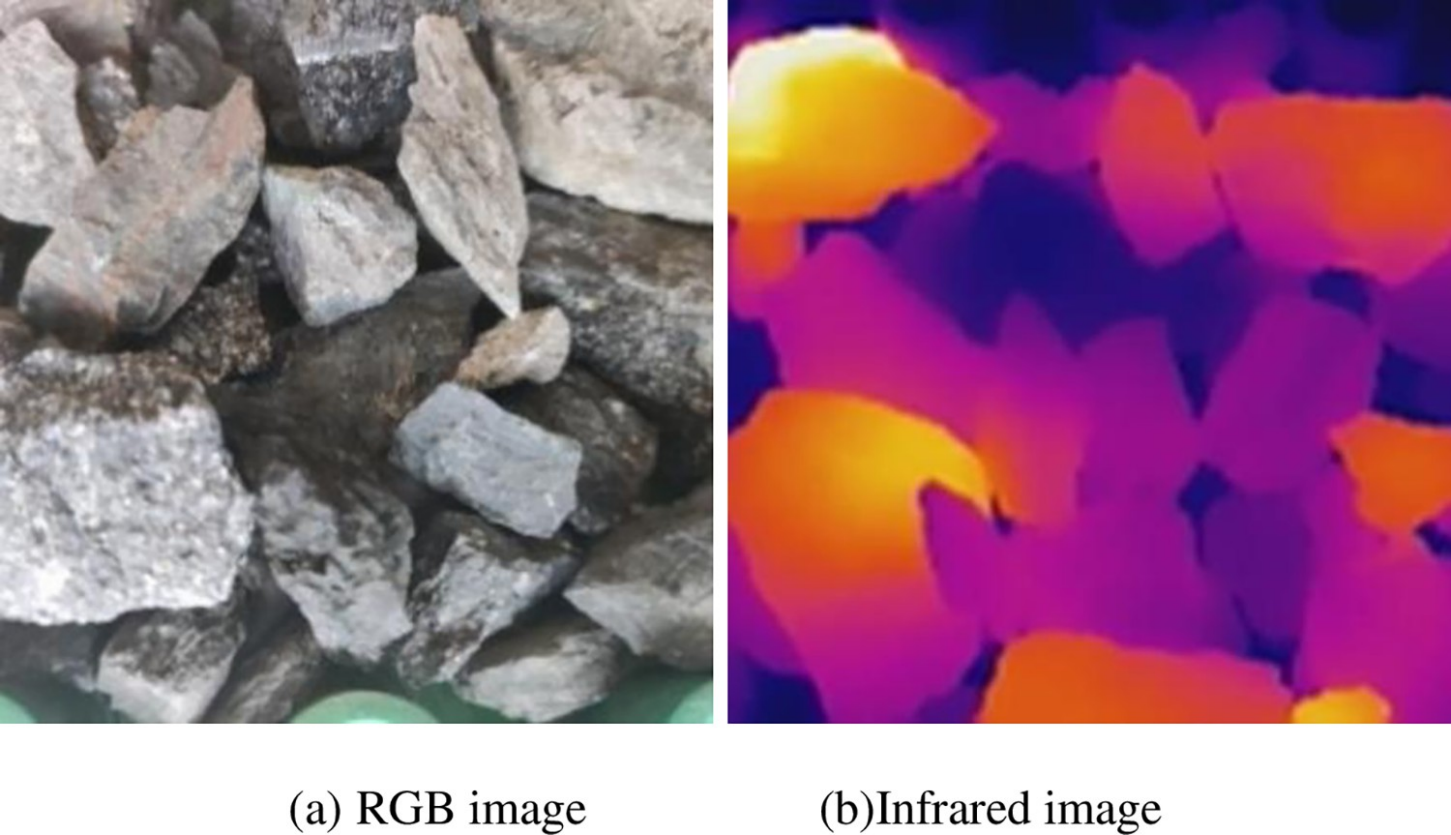
Multimodal image of whole gangue. (a) RGB image; (b) Infrared image.

**Fig 12:**
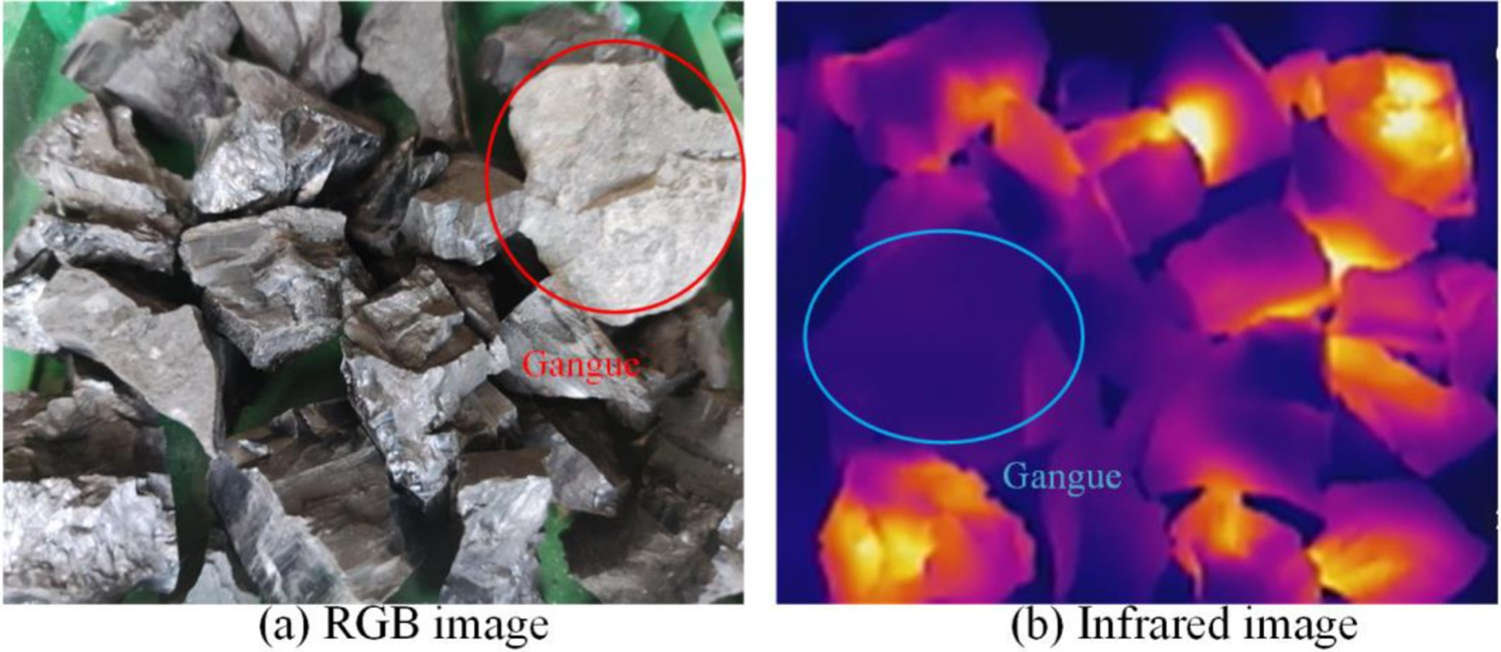
Multimodal image with 10% gangue content. (a) RGB image; (b) Infrared image.

**Fig 13:**
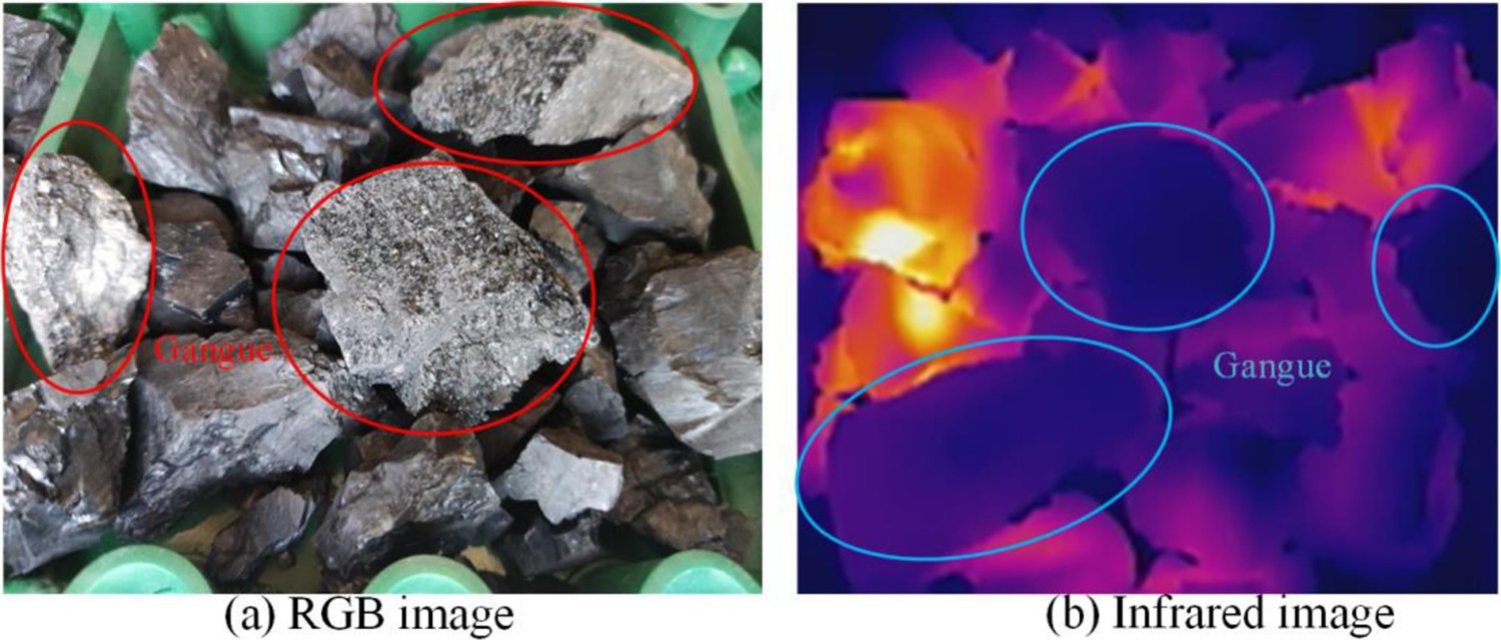
Multimodal image with 25% gangue content. (a) RGB image; (b) Infrared image.

**Fig 14:**
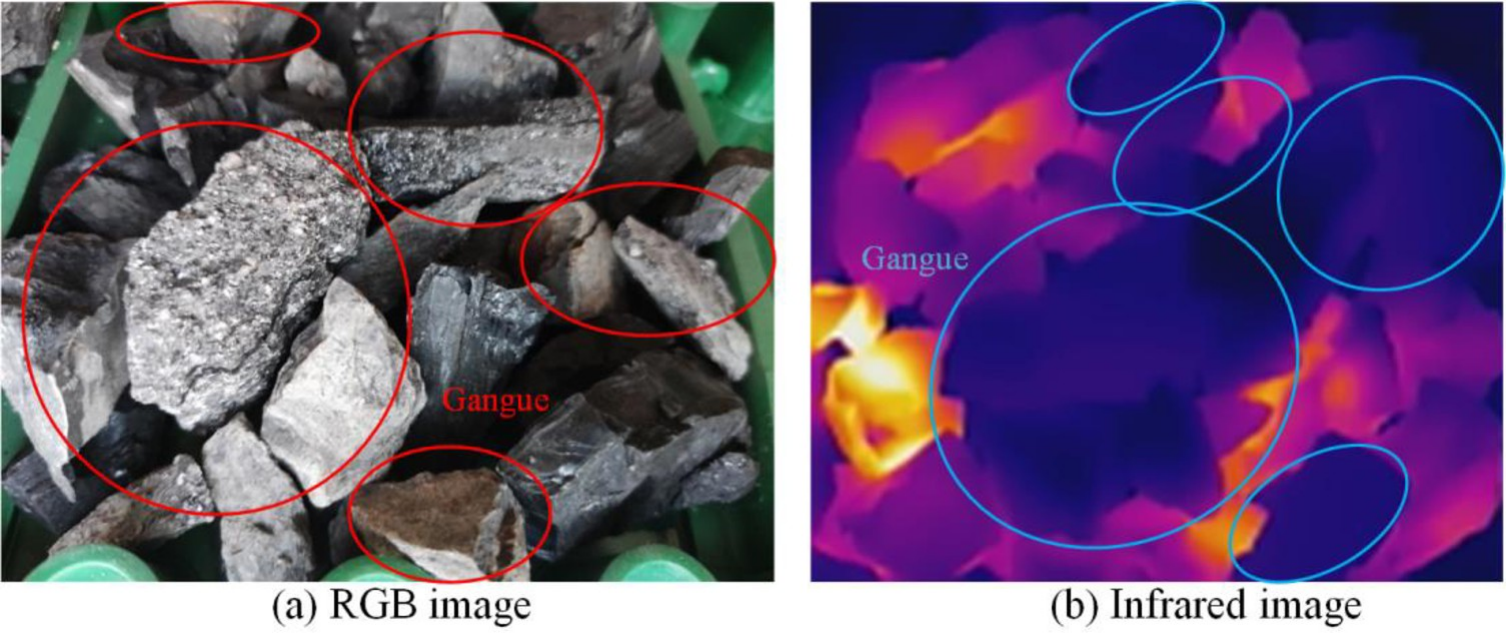
Multimodal image with 50% gangue content. (a) RGB image; (b) Infrared image.

Put the gangue in the coal falling area of the conveyor and run the conveyor. Start the microwave heating system, and simultaneously place the RGB image camera and infrared thermal imager at symmetrical positions on the left and right sides of the conveyor and start them synchronously. After the conveyor runs for 5 min, RGB and infrared video data are obtained, and 7200 RGB images and 6000 infrared images are obtained after decomposing the video data by frames. The video data obtained through five groups of experiments with different mixing rates were copied to the computer and intercepted in the same way, with 36000 RGB images and 30000 infrared images obtained. The multimodal images of the samples obtained are shown in Figs 10–14. The figures show that the whole coal and gangue excited by microwave exhibit a state of near full red in the infrared images, and the brightness of the two images (RGB and IR) is slightly different. The infrared image of the gangue containing sample is in a bright state in the coal block area, while the gangue area is in a dark state. The positional relationship of the IR image is consistent with that of the RGB image.

## 4. Model comparison, validation, and analysis

Under complex and harsh conditions such as low brightness, high dust, and coal gangue stacking, it is difficult to improve the recognition accuracy of coal gangue using a single feature. In this section, the obtained multimodal features of coal gangue were trained and tested on different network models to analyze their recognition performance. Then, the optimal fusion scheme was obtained by different modal feature fusion methods.

### 4.1 Comparison of different single-mode models

According to the classical image classification network commonly used by a CNN, single-mode classification analysis is performed for RGB and infrared modal images. The early training classification networks of AlexNet, VGG-16, and ResNet-18 are built using Python 3.7 language, python architecture, and the PyCharm platform. The number of random samples for each type of single-mode model is 6000, and the total number of samples is 30000. The training and verification sets have a ratio of 4:1, and the number of iterations is set to 400. Other parameter settings are consistent. The training parameter settings are shown in Table 3, and the computer configuration and relevant application versions are shown in Table 4. Through the operation analysis of the Adam optimizer, the recognition ability of the RGB and infrared modal models obtained by the Tensorboard platform is shown in Table 5.

**Table 3:** Training parameters.

Parameter	Numeric value
Learning rate	0.0001
Smooth constant β1	0.9
Smooth constant β2	0.999
Eps	1e-08

**Table 4:** Computer configuration and relevant application versions.

Name	Model version
Central processing unit	AMD Ryzen 9 5950X 16-Core
Graphics processing unit	NVIDIA GeForce RTX 3060
System memory	64G
Solid State Disk	1T
PyCharm	2020.1 x64
Python	3.8.13
Tensorboard	2.9.0

**Table 5:** Recognition and comparison of different models of RGB and infrared modes.

Model of cognition	RGB modality of average recognition rate (%)	Infrared modality of average recognition rate (%)	Time consumed by RGB modality (min)	Time consumed by infrared modality (min)
AlexNet	74.5	79.1	10.34	10.43
VGG-16	31.4	56.8	50.40	47.25
ResNet-18	83.9	87.7	10.58	11.15

Table 5 shows that the training time of the same mode on different networks is different. The time consumed on the AlexNet network is the shortest, whereas the time consumed on the VGG-16 network is the longest. This is because the AlexNet network depth is 8, and the required parameters are the least, whereas the VGG-16 network depth is 16, and its parameters are large. The average recognition rates of RGB and infrared modal data on the ResNet-18 network are 83.9% and 87.7%, respectively. The performance of these two modal data on the ResNet-18 network is higher than that of other networks. This is because this network introduces residual blocks, which solves the degradation problem in the deep network. On the same network model, the recognition accuracy of infrared modal data is higher than that of RGB modal data, indicating that its data resolution characteristics are strong and that it has obvious advantages over RGB modal data.

### 4.2 Fusion results and evaluation

**Fig 15:**
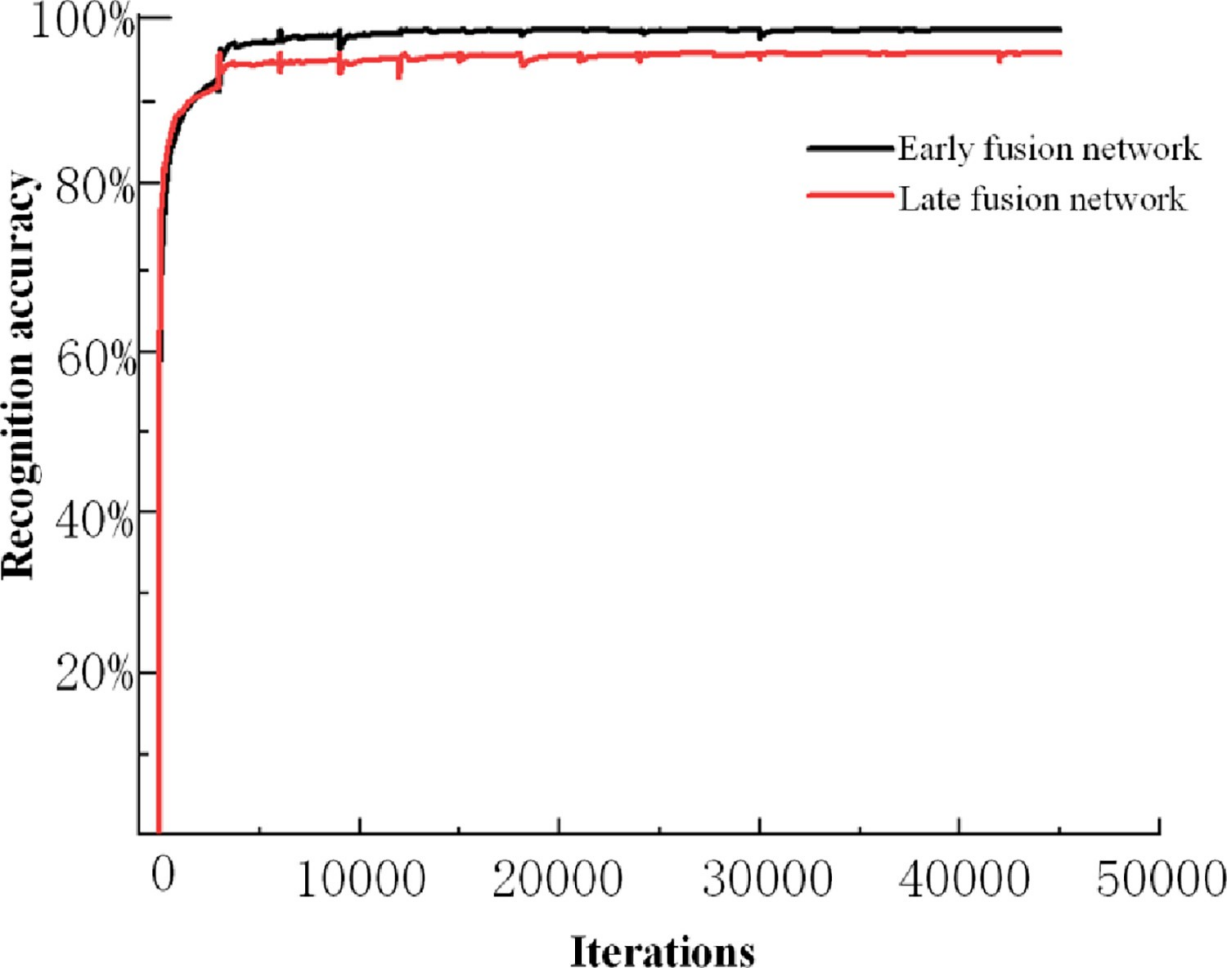
Training recognition rate.

**Fig 16:**
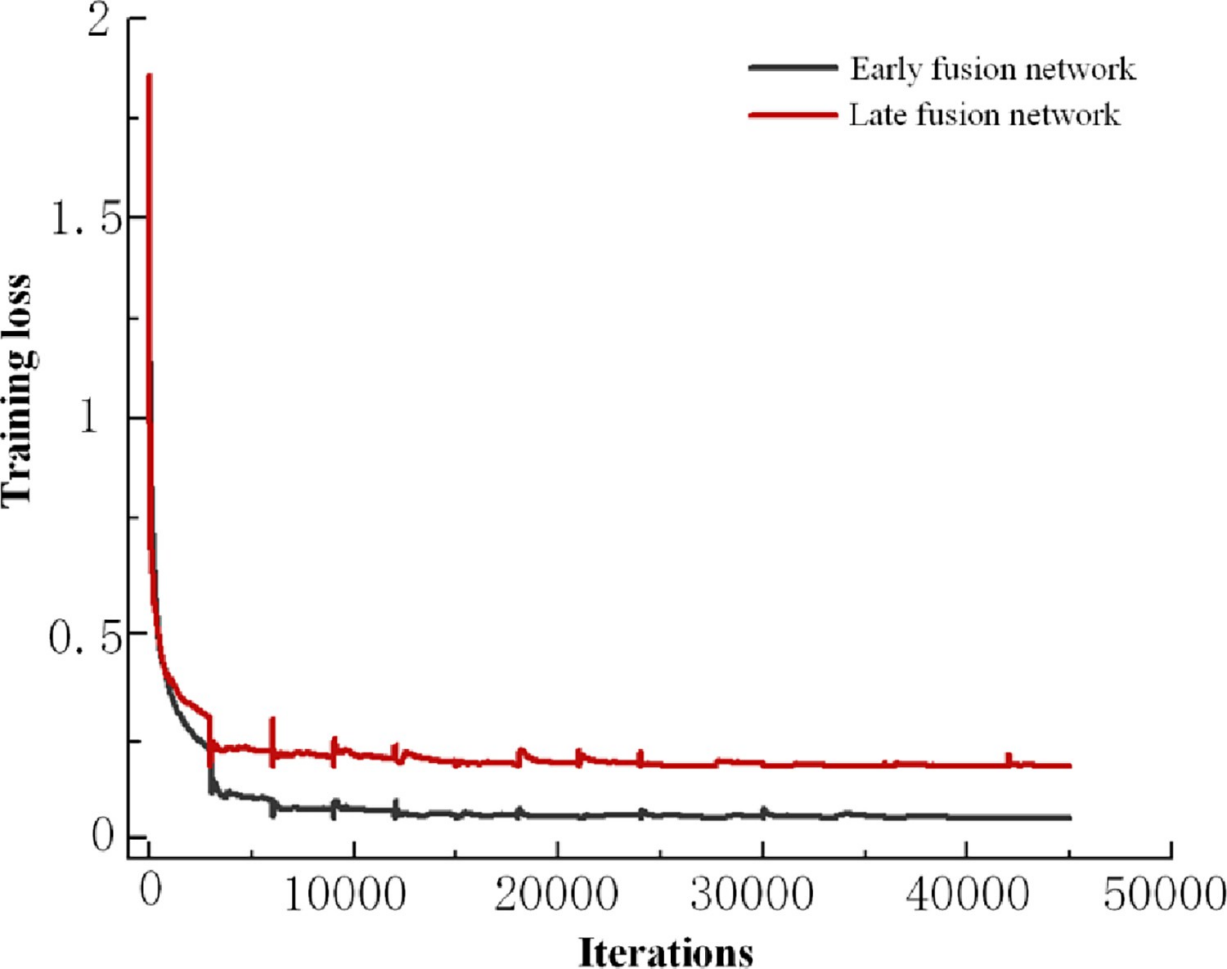
Training loss.

60000 sample data points were obtained through the model information collection platform, each sample type has 12000 data points, and each category contains 6000 data points of infrared and RGB modal data. After dividing the data into the training and test sets in a ratio of 4:1, the two modal image types are input into the feature-based early fusion network and the late fusion network in one-to-one correspondence. The recognition rate of the obtained training process is depicted in Fig 15, and the training loss is shown in Fig 16.

Fig 15 shows that the recognition rate of the two fusion networks increases rapidly with an increase in the number of iterations. After reaching a certain number of iterations, the recognition rate increases slowly. The last two fusion networks are in convergence. The recognition accuracy of the early fusion network is 97.6%, and the recognition accuracy of the late fusion network is 94.4%. This is because the early fusion network performs feature fusion before the input network, and the entire network training process requires fewer parameters, which is easy to converge and has high stability. The late fusion network performs feature fusion after the input network, and its parameters are twice as large as those of the early fusion network. The network training time is long, the stability is low, and the generalization ability is weak. The early fusion network outperforms the late fusion network for the coal gangue classification problem and its samples of RGB and infrared mode fusion.

Fig 16 shows that the training loss of the two fusion networks decreases rapidly with an increase in the number of iterations, and the loss decreases slowly after reaching a certain number of iterations. Finally, the last two fusion networks are in a convergence state. After the number of iterations of the early fusion network reaches 30000, the training loss tends to be stable without large fluctuations. Even if the late fusion network reaches 40000 iterations, the later training loss also exhibits significant fluctuation. The training loss of the early fusion network is 0.1, and the recognition accuracy of the late fusion network is 0.23. The early fusion network can complete the coal gangue classification target task faster and better than the late fusion network.

**Fig 17:**
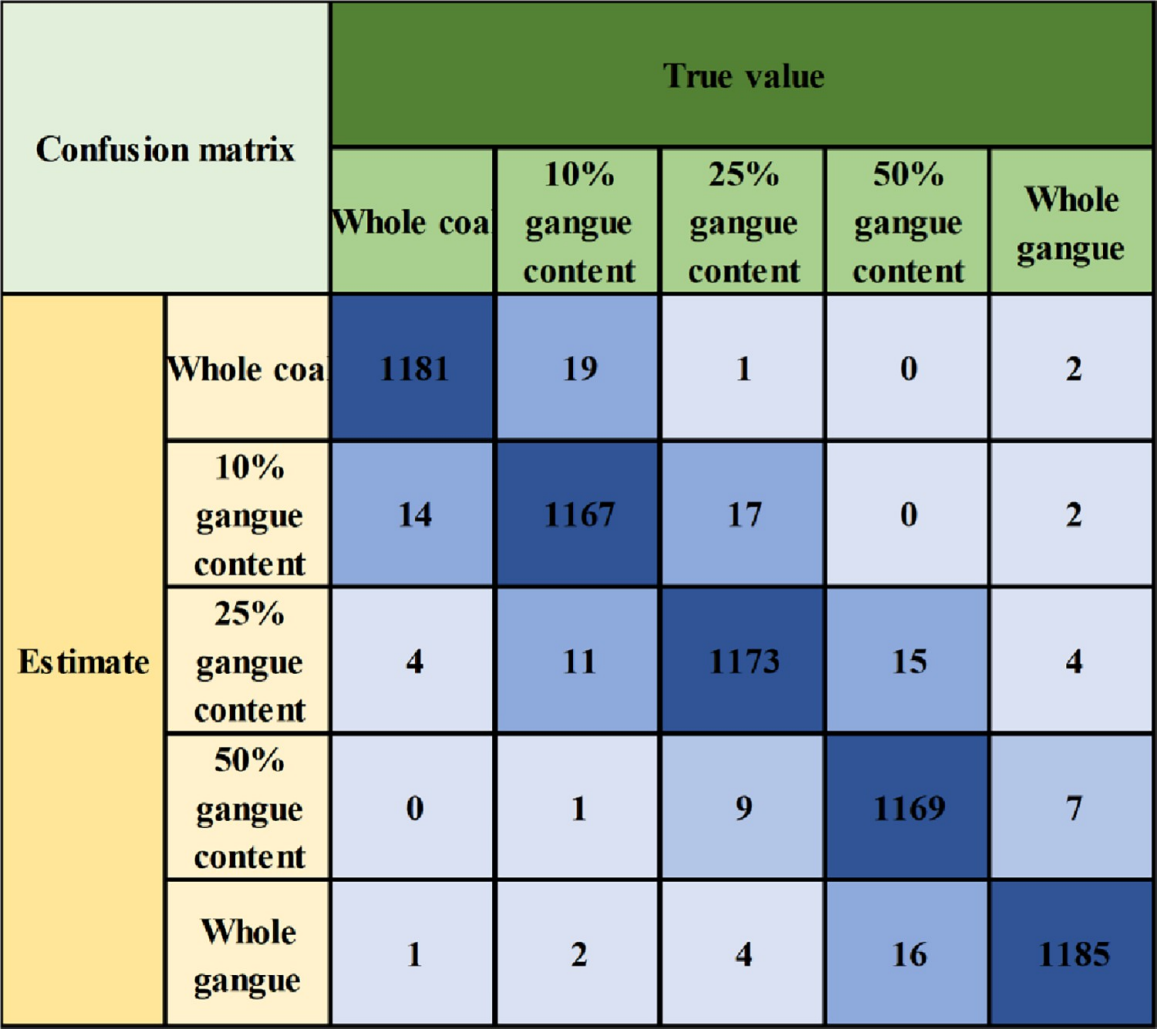
Confusion matrix.

To further analyze the recognition and classification effect of coal gangue, the confusion matrix is used for visual analysis, and the results are shown in Fig 17. As shown in the figure, the total number of samples in the test set is 60,00. Taking the gangue rate of 25% as an example, the total number of samples under this category is 1200, the correct number of samples is 1173, and 27 data samples are misjudged. This is because the colors of some gangue samples are similar to those of coal blocks, and the image of the gangue rate at a certain time is the same as that of other categories, resulting in interference with the classification results, However, such interference only accounts for 2.25% of the total samples. Through Eqs (6)–(11), the accuracy of this category is determined as 97.75%, the precision is 97.18%, and the recall rate is 97.43%, which reflect the sample category status and can ensure high-precision classification results. Similarly, the accuracy of the modal fusion network model is determined as 97.92%, the precision is 97.85%, the recall rate is 97.85%, and the F_1_ score is 97.85%. The early modal fusion method based on ResNet is more stable when classifying and recognizing coal gangue images and can accurately identify different gangue rates.

### 4.3 Analysis of fusion results of different models

To verify the reliability of this model in coal gangue recognition, it is compared with the early fusion methods of other models, as shown in Table 6. The evaluation indicators in the table are obtained by taking the category of 25% gangue as an example. As shown in the table, the ResNet model fusion method is the best. Compared with VGG-16 model fusion, the accuracy of our proposed method is improved by 49.33%. Compared with the AlexNet model fusion method, the proposed method is also significantly improved. The above experimental analysis results show that under the same working and sample data conditions, the coal gangue recognition performance of the proposed ResNet early fusion model is better than that of other model fusion methods.

**Table 6:** Fusion results of different models.

Method	accuracy	Precision	Recall rate	F_1_-Score
VGG early fusion	48.59	47.90	48.14	48.02
AlexNet early fusion	82.25	81.88	82.21	82.04
ResNet early fusion	97.92	97.85	97.85	97.85

## 5. Conclusions

Aiming at the problems of low recognition accuracy of coal gangue and difficult recognition of gangue content rates, through the early fusion of dual ResNet models, a coal gangue recognition method based on image multimodality is proposed in this study. First, the captured video data are preprocessed by frame capturing and clipping. Second, the obtained infrared and RGB image features are extracted for channel fusion. Finally, the fused data are input into the ResNet network for training and classification for an improved recognition effect.

The validity of the proposed method is verified by comparing its results with the single-mode experimental results. In addition, from the analysis of different model fusion results, the ResNet early model fusion network has the highest recognition rate, and the recognition accuracy of gangue content can reach 97.92%.

The proposed method, based on RGB and infrared fusion, has the capability of automatic recognition of coal gangue, which is the basic premise for realizing intelligent coal caving. In the future, relying on gangue recognition technology, the mechanism of the intelligent opening and closing of the tail beam caving will be investigated to achieve the maximum top coal extraction rate. In addition, such a study provides technical support for realizing a digital twin of "three machines and one frame" in coal mines. Through the promotion of gangue recognition and intelligent coal drawing technology, unmanned and intelligent mining of gangue will be realized.
